# Charge Transport Across Single Molecules at the Metal–Superconductor Interface

**DOI:** 10.1002/smll.202412706

**Published:** 2025-05-10

**Authors:** Lorenz Meyer, Maximilian Kögler, Ankur Das, Jose Luis Lado, Nicolas Néel, Jörg Kröger

**Affiliations:** ^1^ Institut für Physik Technische Universität Ilmenau D‐98693 Ilmenau Germany; ^2^ Department of Applied Physics Aalto University 02150 Espoo Finland

**Keywords:** Andreev reflection, scanning tunneling microscopy and spectroscopy, superconductivity

## Abstract

The understanding of electron transport through single atoms or molecules is important for the progress in realizing molecular electronics. Including a superconducting electrode in such junctions is particularly appealing because of the nondissipative character of the charge flow and the associated opportunity of conceiving low‐loss electronic circuits. Moreover, low‐energy excitations that are visible in the highly resolved spectroscopy of the current contain valuable information on electron pairing and pair breaking interactions. This Perspective discusses the appealing physics underlying the current flow across single atoms and molecules anchored to normal‐metal and superconducting electrodes, unveils open questions, and suggests prospective experiments.

## Graphical abstract

The evolution of Andreev reflection in single‐molecule junctions is probed with the decreasing distance between a normal‐metal tip and a conventional superconductor. Andreev reflection becomes most effective when a molecular orbital straddles the Fermi level. This strategy opens the path to control the intrusion of Cooper pairs into assemblies of quantum magnets.

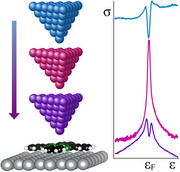

## 1 Introduction

Andreev reflection (AR)^[^ ^1^, ^2^, ^3^ ^]^ is intimately related to the contemporary understanding of charge transport across single atoms and molecules. Experiments that demonstrate this relation require the contact of macroscopic electrodes to the atom or molecule, which was achieved by mechanically controlled break junctions^[^ ^4^, ^5^, ^6^, ^7^ ^]^ and the scanning tunneling microscope (STM).^[^ ^8^, ^9^, ^10^ ^]^ A key finding of these studies is the physical picture of current flow through nanoscale objects. While Ohm's empirical law is not applicable, electron transport channels carry the charge with quantized conductance and transmission probabilities, as is best seen from Landauer's formula^[^ ^11^ ^]^

(1) $$G=\frac{I}{V}=\frac{G_{0}}{2}\sum_{i,j}|\tau_{ij}^{↑}{|}^{2}+{\left|\tau_{ij}^{↓}\right|}^{2}$$

(*I*: current, *V*: bias voltage, G_0_ = 2e^2^/h: quantum of conductance, e: elementary charge, h: Planck constant) where $|\tau_{ij}^{↑,↓}{|}^{2}$ denote the spin‐dependent transmission probabilities of an electron incident on the contact mode *i* and transmitted into mode *j*. The conductance quantization arises due to the junction constriction on the order of the Fermi wavelength of the electron. A special set of electron transport channels are the eigenchannels, which do not mix in the scattering region of the quantum contact and whose transmission probabilities $|T_{i}^{↑,↓}|$ are given by $|T_{i}^{↑,↓}|\delta_{ij}={\left|\tau_{ij}^{↑,↓}\right|}^{2}$.^[^ ^12^, ^13^ ^]^ A pioneering break‐junction study unveiled a direct link between the transport channels and atomic valence orbitals by exploring multiple AR in single‐atom contacts with two superconducting electrodes.^[^ ^14^ ^]^ Multiple AR was also used in a superconducting STM junction to identify the electron transport channels and their transmission for a single C_60_ molecule.^[^ ^15^ ^]^ More recently it was shown for superconducting single‐Al STM contacts that multiple AR affects the junction current also for |*V*| > Δ/e (2Δ: width of the Bardeen‐Cooper‐Schrieffer (BCS) energy gap of the superconductor (SC)) and the information on transport channel transmission can be extracted from the excess current.^[^ ^16^ ^]^

**Figure 1:**
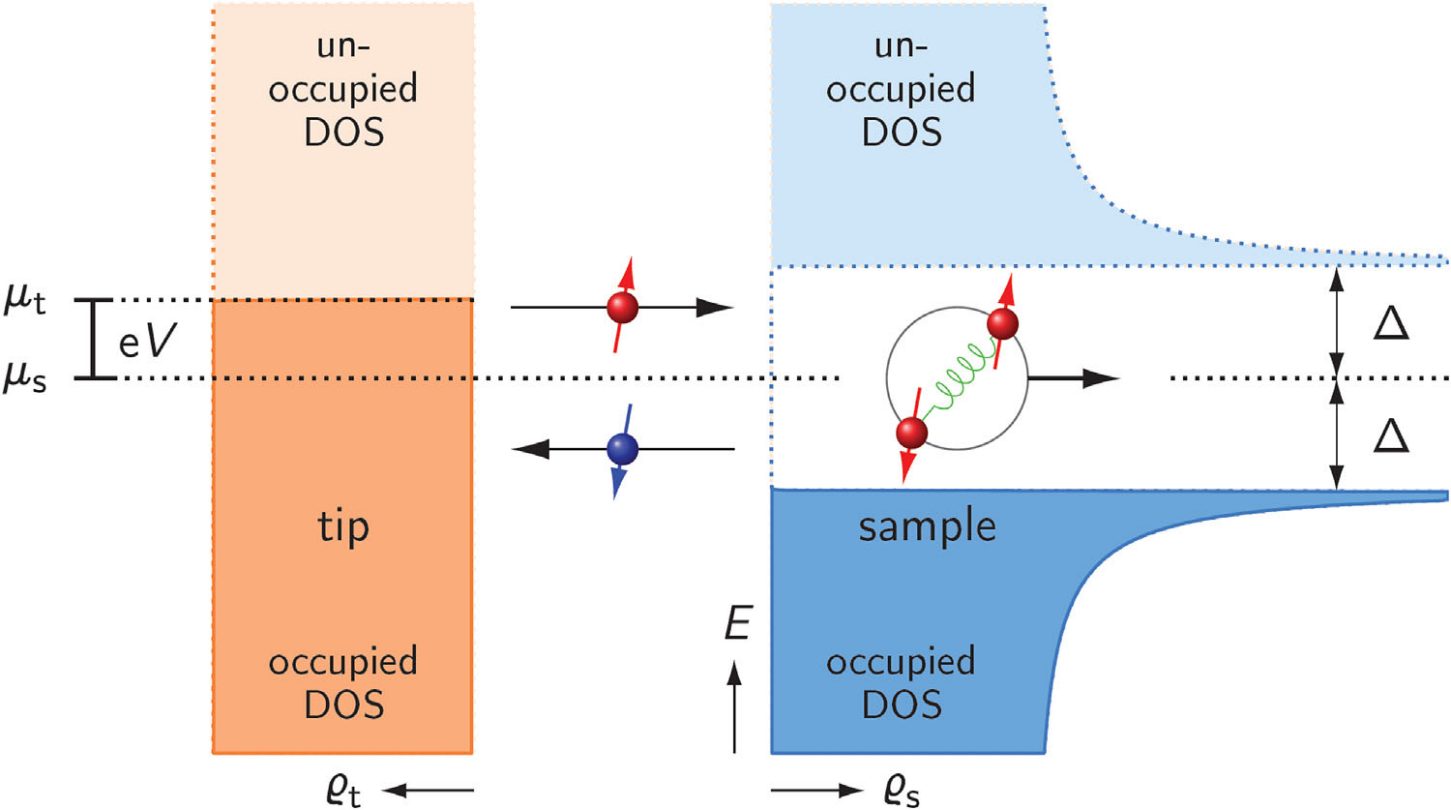
Schematic illustration of Andreev reflection at the interface between a normal‐metal tip and a superconductor sample. For simplicity, the density of states (DOS) of the tip, ϱ_t_, is assumed constant, while the DOS of the sample, ϱ_s_, is defined by the superconducting energy gap of width 2Δ at zero temperature. The applied bias voltage *V* raises the chemical potential of electrons from µ_s_ to µ_t_ = µ_s_ + e*V* < Δ. An electron with spin up and energy *E*_e_ = µ_t_ is retroreflected as a hole with spin down and energy *E*_h_ = µ_s_ − e*V* giving rise to a Cooper pair in the superconductor at µ_s_.

The process of AR at the interface between a normal conductor (NC) and an SC is of particular interest in this Perspective. **Figure** **1** illustrates the situation for an NC tip of an STM and an SC at vanishing temperature for simplicity. Electrons of the NC tip with energy |e*V*| < Δ do not find unoccupied quasielectron states in the SC and, thus, are impeded to cross the junction, they are reflected at the interface. The AR scenario, however, suggests a transport mechanism where the NC electron impinges on the NC–SC interface and couples with a second electron of opposite spin to a Cooper pair. The latter can propagate within the BCS energy gap of the SC. To match charge, spin, momentum, and energy conservation in AR, a hole of opposite spin and energy has to be retroreflected in the NC upon Cooper pair transmission into the SC. In more detail, the second electron originates from the occupied electron states of the NC and leaves behind a hole that travels with a momentum opposite to the momentum of the incident electron, which is therefore referred to as retroreflection, as opposed to ordinary reflection. Because time reversal symmetry applies, AR likewise works for a Cooper pair moving toward the NC–SC interface: an incident hole of the NC propagating towards the NC–SC interface is retroreflected as an electron. In other words, the Cooper pair electrons are separated into the retroreflected electron and the electron occupying the hole state. The net charge transferred per AR is 2e, which is entailed by a current at |*V*| < Δ/e that is two times as large as the current expected from the transport of a single electron; this current due to AR is hence referred to as excess current.^[^ ^17^ ^]^

For quantum point contacts forming an NC–SC interface, AR can be described by the Blonder–Tinkham–Klapwijk (BTK) model.^[^ ^18^, ^19^ ^]^ A prerequisite for its applicability is the variation of the electric potential and the BCS energy gap to their full asymptotic values on a scale shorter than the SC coherence length ξ. An important ingredient of the model is the separation of the NC and SC by an adjustable barrier in order to consider, e. g., realistic oxide films or different Fermi velocities of the materials, which may cause ordinary electron reflection and a reduction of AR. This barrier is described by a Dirac‐δ‐function potential located at the NC–SC interface (*x* = 0) of the form *H*δ(*x*) with the dimensionless barrier strength *Z* = *H*/(ℏ*v*_F_) (ℏ = h/(2π), *v*_F_: Fermi velocity), which determines the BTK transmission as τ = 1/(1 + *Z* ^2^).

Andreev reflection is at work in the proximity effect, which describes the diffusion of Cooper pairs across the NC–SC interface into the NC material, that is, the Cooper pair density of the SC is finite also in the NC on a length scale defined by ξ.^[^ ^20^, ^21^, ^22^, ^23^ ^]^ Another interesting application of AR is its use in exploring the spin polarization of a current across the NC–SC interface. According to Figure 1, the incident electron and retroreflected hole have opposite spin. If the NC material exhibits an imbalance between spin‐up and spin‐down states, e. g., in the case of ferromagnetism, a suppression of AR may be expected. A reduction of AR in the presence of a spin‐polarized current was indeed experimentally observed.^[^ ^24^, ^25^, ^26^, ^27^ ^]^

Recently, AR has again attracted substantial attention for the description of charge transport across the ferromagnet–SC interface,^[^ ^28^, ^29^ ^]^ where spin‐orbit coupling supports spin‐triplet superconductivity.^[^ ^30^, ^31^ ^]^ The latter is supposed to be crucial for superconducting spintronics^[^ ^32^ ^]^ and topologically protected Majorana bound states.^[^ ^33^, ^34^ ^]^

A very new class of magnetically ordered materials – altermagnets^[^ ^35^, ^36^ ^]^ – has just started to enter into the focus of research. They are characterized by a zero net magnetization, as observed for antiferromagnets, and, at the same time, a spin‐split band structure, like in ferromagnets. Owing to the spin‐resolved *d*‐wave symmetry of the altermagnet band structure,^[^ ^37^ ^]^ AR at the altermagnet–SC interface is determined by the orientation of the altermagnet, and the momentum‐dependent spin polarization of the altermagnetic state can be probed by AR conductance spectroscopy.^[^ ^37^, ^38^, ^39^, ^40^ ^]^

In this Perspective, the focus is on STM experiments contributing to the understanding of AR. The motivation is twofold. First, despite the importance of AR, STM studies of charge transport across the NC–SC interface are surprisingly scarce. Second, in contemporary condensed‐matter physics it has become evident that a microscopically substantiated rationale to low‐energy excitations in superconducting junctions is mandatory for a thorough interpretation of zero‐bias anomalies in *I*–*V* characteristics.

## 2 Experimental Evidence from Scanning Tunneling Spectroscopy for Andreev Reflection

The BTK model has proved very successful in the description of charge transport across NC–SC point contacts^[^ ^4^ ^]^ and quantum dots.^[^ ^41^ ^]^ The first STM experiment unveiling AR showed a zero‐bias resonance (ZBR) in spectra of the differential conductance (d*I*/d*V*) observed from low‐ohmic junctions comprising Au or Pt‐Rh tips and a polycrystalline Pb substrate.^[^ ^42^ ^]^ For a cuprate high‐temperature superconductor, AR was likewise reported for an STM junction comprising a Pt‐Ir tip and the (110) crystal face of YBa_2_Cu_3_O_7 − δ_.^[^ ^43^ ^]^ The ZBR in d*I*/d*V* spectroscopy was rationalized as the signature of AR. To this end, the BTK model was generalized to *d*‐wave pairing. Surprisingly, the next STM experiment addressing AR was performed more than one decade later, where a W tip was approached to the A15‐compound SC V_3_Si(100) up to and beyond contact.^[^ ^44^ ^]^ In these studies, rather than creating a low‐ohmic junction^[^ ^42^ ^]^ with considerable damage to the tip and surface structure, the junction conductance was carefully changed on the order of the quantum of conductance, which ensured the invariance of the tip and sample structural integrity. Importantly, even at the highest junction conductance probed in these experiments (≈6 G_0_; if not otherwise stated, the junction conductance is always set for sample voltages outside the d*I*/d*V* signature of the BCS energy gap), AR appeared at best as the incomplete filling of the BCS energy gap with spectral weight, rather than as a fully developed ZBR. A noncontact approach to AR was put forward in a recent STM work,^[^ ^45^ ^]^ where the exponential evolution of the d*I*/d*V* signal with the tip–surface separation was compared for electron energies inside and outside the BCS energy gap of Pb(110). The stronger exponential increase observed from d*I*/d*V* for voltages |*V*| ⩽ Δ/e than for |*V*| > Δ/e with decreasing tip–surface distance was traced to AR. This method was later applied to the unconventional superconductor FeSe.^[^ ^46^ ^]^

**Figure 2:**
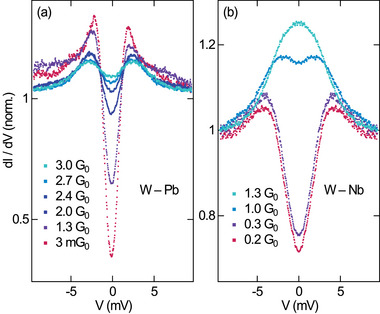
Comparison of the evolution of the BCS energy gap with increasing (from bottom to top) junction conductance for a (a) W–Pb and a (b) W–Nb (reproduced with permission,^[^ ^47^ ^]^ copyright 2017, American Physical Society) contact. The junction conductances were set at the voltages a) 15 mV, b) 10 mV, which are identical to the feedback loop parameters for the spectra. The d*I*/d*V* spectra were acquired at 4.8 K with an ac modulation of a) 100 µV_rms_, b) 250 µV_rms_, and are normalized to their value at a) 15.0 mV, b) 9.5 mV.

A situation similar to V_3_Si(100) is encountered for a Pb(111) surface forming an STM junction with a W tip. **Figure** **2**a shows that the emerging of AR at the atomic‐scale W–Pb contact is apparently impeded even for an elevated junction conductance of 3 G_0_. In contrast, a recent STM experiment exploring the evolution of AR in junctions comprising a W tip and a Nb(110) surface^[^ ^47^ ^]^ revealed that the ZBR associated with AR was completely reached already at a junction conductance of ≈1.3 G_0_ (Figure 2b), which is clearly at odds with the findings for V_3_Si(100)^[^ ^44^ ^]^ and Pb(111) (Figure 2a).

The intimately related question as to why V_3_Si(100) and Pb(111) on the one hand and Nb(110) on the other hand behave differently in the evolution of AR has not been answered yet. Before addressing this open question, three possible rationales one may be tempted to resort to are discussed.

For single‐atom contacts, the maximum number of electron transport channels is determined by the number of valence orbitals,^[^ ^14^ ^]^ which for the *sp*‐metal Pb is 4 and for the *d*‐metal Nb is 5. Indeed, break junction experiments revealed subharmonic fine structure in the BCS energy gap due to multiple AR in Pb^[^ ^14^ ^]^ and Nb^[^ ^14^, ^48^ ^]^ junctions, which could best be described by the contribution of, respectively, 4 and 5 transport channels with different transmission probabilities. If all transport channels of Pb were transmitting with unity probability, a total conductance of the junction of 4 G_0_ would therefore be expected. Consequently, at first sight, the partial suppression of AR at a junction conductance of 3 G_0_ (Figure 2a) would be in line with the deviation from the ideal junction transmission. However, this reasoning is not applicable to Nb single‐atom junctions where the fully transparent contact would give rise to a conductance of 5 G_0_. Although staying clearly below 5 G_0_, AR is completely developed at already 1.3 G_0_ (Figure 2b).

Another possible scenario traces the observed differences to a strong mismatch in Fermi velocities *v*_F_ of the materials forming the interface. Indeed, the BTK parameter *Z* defining the strength of the barrier at the NC–SC interface with different $v_{F}^{\text{NC}}$ and $v_{F}^{\text{SC}}$ adopts the form of an effective barrier strength $Z^{*}=\sqrt{Z^{2}+{\left(1-\nu_{F}\right)}^{2}/4\nu_{F}}$ where ν_F_ is the ratio of Fermi velocities.^[^ ^18^ ^]^ For Pb and Nb (**Table** **1**), a nearly‐free‐electron approximation gives rise to $v_{F}^{\text{Pb}}\approx 1.8\cdot 10^{6}\;ms^{-1}$ and $v_{F}^{\text{Nb}}\approx 1.4\cdot 10^{6}\;ms^{-1}$.^[^ ^51^ ^]^ The strongly anisotropic Fermi surfaces of W and V_3_Si are entailed with a range of *v*_F_, which complicates a comparison. For W, $0.5\cdot 10^{6}\;{\mathrm{ms}}^{-1}\leq v_{F}^{W}\leq 1.5\cdot 10^{6}\;{\mathrm{ms}}^{-1}$,^[^ ^54^, ^55^, ^56^ ^]^ while for V_3_Si, $0.02\cdot 10^{6}\;{\mathrm{ms}}^{-1}\leq v_{F}^{V_{3}\text{Si}}\leq 0.9\cdot 10^{6}\;{\mathrm{ms}}^{-1}$ ^[^ ^49^ ^]^ was reported. One may therefore tentatively conclude that the combination of W tips and Pb, Nb substrates would lead to a more pronounced evolution of AR than the W–V_3_Si combination. However, Figure 2 clearly shows that this conclusion does not apply to W–Pb and W–Nb junctions because despite a similar ν_F_, AR progresses in a vastly different manner in these two contacts.

**Table 1:** Collection of Fermi velocities *v*_F_ and coherence lengths ξ for the indicated materials.

Substrate	*v*_F_ ($10^{6}\;ms^{-1}$)	ξ [nm]
V_3_Si	0.02–0.9^[^ ^49^ ^]^	≈6^[^ ^50^ ^]^
Pb	≈1.8^[^ ^51^ ^]^	51–90^[^ ^52^, ^53^ ^]^
Nb	≈1.4^[^ ^51^ ^]^	≈41^[^ ^52^ ^]^
W	0.5–1.5 ^[^ ^54^, ^55^, ^56^ ^]^	–

In addition, the superconducting coherence lengths of the materials are not conclusive for rationalizing the observations. One could argue that a small coherence length requires a small distance between the NC tip and the SC and therefore a high junction conductance for clearly seeing the AR‐associated ZBR in d*I*/d*V* spectra. While for V_3_Si with ξ ≈ 6 nm^[^ ^50^ ^]^ (Table 1) this conjecture would apparently hold, it is however falsified by the coherence lengths of Pb (51 nm ⩽ ξ ⩽ 90 nm)^[^ ^52^, ^53^ ^]^ and Nb (ξ ≈ 41 nm).^[^ ^52^ ^]^

**Figure 3:**
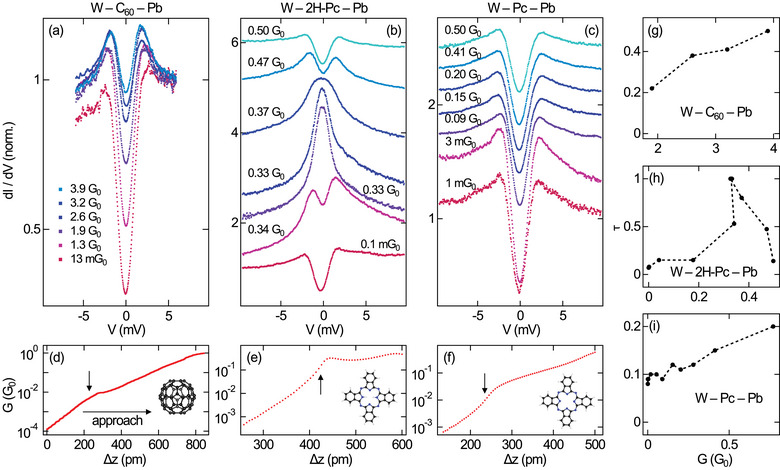
Evolution of the Pb(111) BCS energy gap in single‐molecule STM junctions comprising a W tip and a (a) C_60_, (b) 2H‐Pc (reproduced with permission,^[^ ^57^ ^]^ copyright 2025, American Physical Society), c) Pc (reproduced with permission,^[^ ^57^ ^]^ copyright 2025, American Physical Society) molecule with increasing (from bottom to top) conductance, which was set at sample voltages outside the BCS energy gap: a) 10 mV, b,c) 20 mV. Feedback loop parameters: 10 mV (C_60_), 20 mV (2H‐Pc, Pc). The spectra are normalized to unity at 5 mV (C_60_), −15 mV (2H‐Pc, 0.1 mG_0_–0.33 G_0_), 15 mV (2H‐Pc, 0.37 G_0_–0.50 G_0_), 15 mV (Pc). d–f) Conductance *G* of the junctions in (a)–(c) as a function of tip excursion Δ*z*. The *G* traces were recorded at 10 mV (C_60_) and 20 mV (2H‐Pc, Pc). The approach direction is marked by the horizontal arrow in (d), which applies to (e), (f), too. Vertical arrows mark the tunneling‐to‐contact transition. Insets to (d)–(f): Sketches of the respective molecules probed in the contact experiments. g–i) BTK transmission τ as a function of the junction conductance *G* evaluated at g) 10 mV and h,i) 20 mV.

At present, the findings for the clean surfaces remain elusive, but a possible underlying mechanism is provided by the analysis of charge transport across single‐molecule junctions. **Figure** **3** compares the evolution of the Pb(111) BCS energy gap in d*I*/d*V* spectroscopy with a W tip approaching a C_60_ exposing a C pentagon to the tip (Figure 3a), a phthalocyanine (C_32_H_18_N_8_, 2H‐Pc, Figure 3b),^[^ ^57^ ^]^ and its derivative Pc (Figure 3c),^[^ ^57^ ^]^ which results from 2H‐Pc by the on‐surface abstraction of the two pyrrolic H atoms using electron injection from the tip.^[^ ^58^, ^59^, ^60^, ^61^, ^62^ ^]^ As observed for pristine Pb(111), AR is similarly weak for C_60_ on Pb(111). The BCS energy gap region is filled with spectral weight with increasing (from bottom to top) junction conductance. However, the gap is clearly visible even at the elevated conductance of 3.9 G_0_, which belongs to the deep contact range. Figure 3d shows in a logarithmic presentation the evolution of *G* with the tip excursion. The arrow marks the transition from tunneling to contact where a deviation from the uniform exponential increase of *G* occurs, which signals the collapse of the tunneling barrier.^[^ ^8^, ^9^, ^10^ ^]^

In contrast, a strong ZBR develops for the single‐2H‐Pc junction on the same surface at already appreciably lower *G* (Figure 3b).^[^ ^57^ ^]^ Remarkably, for 2H‐Pc the ZBR attenuates again with increasing *G*. This observation is at odds with the BTK picture of AR where an increasing (decreasing) junction transmission is associated with the enhancement (degradation) of AR. Consequently, an additional mechanism that has hitherto been ignored must be operative in enhancing and degrading AR. A first hint to this mechanism is provided by the *G*‐versus‐Δ*z* trace of the 2H‐Pc junction (Figure 3e) where the transition from tunneling to contact is accompanied by a rapid rise (arrow) and followed by a shallow minimum. This peculiar behavior of a nonmonotonous variation of *G* with increasing Δ*z*, which contrasts the observed monotonous increase of *G* for the C_60_ (Figure 3d) and the Pc (Figure 3f) junction, can be simulated by a 2H‐Pc molecular orbital (MO) that concomitantly with tip approach shifts towards and across *E*_F_ (see below).^[^ ^57^ ^]^ In a similar conductance range, the d*I*/d*V* data of single Pc reveal the nearly undistorted signature of the BCS energy gap (Figure 3c), which is akin to the observations from C_60_.

Before substantiating the presence of a shifting MO via d*I*/d*V* spectroscopy, the evolution of the Pb(111) superconducting energy gap is quantitatively analyzed. The BTK transmission τ was extracted to this end by fitting the temperature‐broadened BTK function to the spectroscopic data.^[^ ^57^ ^]^ In the case of 2H‐Pc, Kondo screening of a finite molecular magnetic moment acquired at tip–molecule contact has to be considered by appropriately adjusting the tip DOS.^[^ ^57^ ^]^ Figure 3g–i shows the resulting τ as a function of *G*. The evolution of τ for the W–2H‐Pc–Pb junction (Figure 3h) differs in two important aspects from the variation of τ for W–C_60_–Pb (Figure 3g) and W–Pc–Pb (Figure 3i) junctions. First, the W–2H‐Pc–Pb junction reaches τ = 1, i. e., the fully developed AR, at 0.33 G_0_, whereas the W–C_60_–Pb contact reaches τ = 0.5 for elevated *G* = 3.9 G_0_ and the W–Pc–Pb junction adopts τ = 0.2 at *G* = 0.8 G_0_. Second, τ exhibits a clearly nonmonotonous behavior, that is, after reaching τ = 1 the BTK transmission decreases again for increasing *G*.

**Figure 4:**
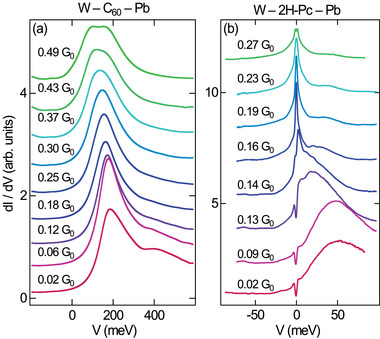
Shift of an unoccupied MO for a) C_60_ exposing a C pentagon to the tip (reproduced with permission,^[^ ^63^ ^]^ copyright 2017, American Physical Society) and b) 2H‐Pc on Pb(111) (reproduced with permission,^[^ ^57^ ^]^ copyright 2025, American Physical Society) approached with a W tip. The indicated junction conductances were set at the same voltages as the feedback loop parameters for spectroscopy: a) 600 mV, b) 100 mV. The spectra are vertically offset.

Spectroscopy of d*I*/d*V* in a wider range of voltages indicates a possible origin of the different AR behavior in the various single‐molecule junctions. **Figure** **4** compares d*I*/d*V* spectra for C_60_ (Figure 4a)^[^ ^63^ ^]^ and 2H‐Pc (Figure 4b)^[^ ^57^ ^]^ on Pb(111) approached with a W tip each. The voltage range was chosen such as to observe the spectroscopic signature of an unoccupied MO in both cases. Spectroscopy of the MO (Figure 4) and of the BCS energy gap (Figure 3) was performed with different tips. However, the correlation of the MO shift with the AR enhancement is independent of the tip.^[^ ^57^ ^]^ The modulation broadening in Figure 4a impedes the appearance of the BCS energy gap in the spectra, while it was sufficiently low in Figure 4b to observe both the energy gap and the MO. In both cases, a shift of an unoccupied MO toward *V* = 0, i. e., to *E*_F_, with increasing *G* is visible. For C_60_, the MO signature starts to weakly overlap with *E*_F_ for *G* > 0.4 G_0_, while the 2H‐Pc MO exhibits appreciable overlap already for *G* > 0.1 G_0_ and straddles the Fermi level at *G* ≈ 0.15 G_0_. This MO energy shift for 2H‐Pc is consistent with the nonmonotonous *G*‐versus‐Δ*z* evolution (Figure 3e).^[^ ^57^ ^]^ The single‐Pc junctions do not evidence MO signatures in an even wider voltage range.^[^ ^57^ ^]^ These results support the idea that AR is efficiently enhanced only in the case of ample electron DOS at *E*_F_, which may be provided by a MO that is well centered at the Fermi level.

**Figure 5:**
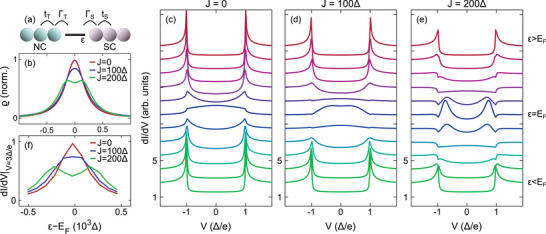
a) Illustration of the one‐dimensional NC–SC junction geometry the simulations are based on. The symbols are explained in the text. b) Calculated spectral density ϱ(ε = *E*_F_) of the MO for the marked intramolecular magnetic exchange energies *J*. c–e) Simulated d*I*/d*V* spectra for different MO energies ε. (f) Evolution of calculated d*I*/d*V* at *V* = 3Δ/e with MO energy ε for different *J*.

This conjecture is further corroborated by model calculations (**Figure** **5**), which have recently been discussed in detail.^[^ ^57^ ^]^ Here, the basic ingredients of the simulations are briefly introduced. The NC–SC junction geometry is one‐dimensional (Figure 5a). Electrons of the SC substrate are described by the Hamiltonian $H_{S}=\mu_{S}\sum_{i,s}c_{i,s}^{†}c_{i,s}+t_{S}\sum_{\langle ij\rangle s}c_{i,s}^{†}c_{j,s}+\Delta \sum_{i}c_{i,↑}^{†}c_{i,↓}^{†}+\text{H.c.}$, while electrons of the NC tip obey $H_{T}=\mu_{T}\sum_{i,s}d_{i,s}^{†}d_{i,s}+t_{T}\sum_{\langle ij\rangle s}d_{i,s}^{†}d_{j,s}+\text{H.c.}$ The Hamiltonian of the molecule takes the form $H_{M}=\varepsilon \sum_{s}f_{s}^{†}f_{s}+J\sum_{s,s^{\prime }}\sigma_{z}^{s,s^{\prime }}f_{s}^{†}f_{s^{\prime }}+\text{H.c.}$ In these Hamiltonians, µ_S_ and µ_T_ define the chemical potentials of substrate and tip, respectively, *t*_S_ and *t*_T_ are the electron hopping parameters, and 〈*ij*〉 indicates the restriction of the sums to nearest neighbors. The operator $c_{i,s}^{†}$ ($d_{i,s}^{†}$) creates an electron at position *i* with spin *s* ∈ {↑, ↓}, while *c*_*i*,*s*_ (*d*_*i*,*s*_) annihilates it; for the molecule, these operators are denoted $f_{s}^{†}$ and $f_{s}$. The *z* Pauli matrix is given by $\sigma_{z}^{s,s^{\prime }}$. An important ingredient of the model is the adjustable single molecular energy level ε. The spectral density of the associated orbital is obtained from $\varrho \left(\varepsilon \right)=-ℑ\{\text{Tr}\left[P_{e}{\left(\varepsilon -H_{M}-\Sigma_{S}-\Sigma_{T}\right)}^{-1}\right]\}/\pi$, where $\Sigma_{\text{S,T}}={\left|\Gamma_{\text{S,T}}\right|}^{2}G_{\text{S,T}}^{\text{surf}}$ are the self‐energies of substrate and tip, $G_{\text{S,T}}^{\text{surf}}$ the surface Green's functions, and $P_{e}$ the projection operator in the particle Nambu sector.^[^ ^64^ ^]^ Figure 5b shows the graphs of ϱ(ε = *E*_F_) for different intramolecular magnetic exchange coupling energies *J*. For *J* = 0, a single Lorentzian is observed, which broadens for *J* = 100 Δ and exhibits a clear exchange splitting for *J* = 200 Δ. The hybridization of the molecular energy level with the two electrodes is modeled as $H_{C}=\Gamma_{S}\sum_{s}f_{s}^{†}d_{0,s}+\Gamma_{T}\sum_{s}c_{0,s}^{†}f_{s}+\text{H.c.}$ with Γ_S_ and Γ_T_ accounting for the coupling strengths of the molecule to the substrate and the tip, respectively. A Green's function method is then applied to the total Hamiltonian $H=H_{S}+H_{T}+H_{M}+H_{C}$.

For different values of ε ranging between ε = 0.5 *t*_S_ > *E*_F_ and ε = −0.5 *t*_S_ < *E*_F_ the d*I*/d*V* spectrum of the DOS close to *E*_F_ is simulated for *J* = 0 (Figure 5c), *J* = 100 Δ (Figure 5d), *J* = 200 Δ (Figure 5e). In good agreement with the experimental data, AR is strengthened when the maxima of ϱ are located at *E*_F_. For the peaklike spectral densities in the cases of *J* = 0 (Figure 5c) and *J* = 100 Δ (Figure 5d) the enhancement of AR can clearly be inferred from the middle spectra of the collections of the simulated data. Previously, a theoretical work that generalized the Landauer formula (Equation 1) for NC–SC quantum point contacts likewise showed the AR enhancement by an electronic resonance at the Fermi level.^[^ ^65^ ^]^ The close inspection of the middle spectrum of Figure 5d shows, however, that the finite intramolecular exchange interaction causes less developed AR compared to the situation with *J* = 0. This attenuation becomes notable for *J* = 200 Δ (Figure 5e). Now, a weak enhancement of AR occurs when each of the exchange‐split orbitals passes through *E*_F_ (4th and 8th spectrum of Figure 5e). With the centroid of ϱ(ε) being located at *E*_F_ (middle spectrum of Figure 5e) AR is weakened again reflecting the maximum spin imbalance of the molecular electronic structure, which is known from spin‐polarized currents across magnetic quantum point contacts.^[^ ^24^, ^25^, ^26^, ^27^ ^]^ Concomitantly, the molecular magnetic moment is maximized, which gives rise to Yu‐Shiba‐Rusinov (YSR) states^[^ ^66^, ^67^, ^68^ ^]^ appearing as pairs of peaks at symmetric energies above and below *E*_F_ inside the BCS energy gap. These intragap states can experimentally be observed in d*I*/d*V* spectra acquired with an SC tip that offers high energy resolution.^[^ ^57^ ^]^ To see the dependence of AR on the MO energy more clearly, Figure 5f presents the variation of simulated d*I*/d*V* at *V* = 3Δ/e, which is well outside of the BCS energy gap and can be compared with the BTK transmission τ. The initial increase and subsequent decrease of d*I*/d*V*|_*V* = 3Δ/e_ for *J* = 0 and *J* = 100 Δ as a function of ε is in accordance with the experimentally observed behavior of τ for 2H‐Pc (Figure 3h). The overall lower value of d*I*/d*V*|_*V* = 3Δ/e_ for *J* = 200 Δ and its double‐peak structure is rationalized in terms of a weakened AR due to an exchange‐split MO.

Before discussing perspective experiments and opportunities relying on the presented results, possible origins of the MO shift are suggested. The gradual shift of the MO is induced by the approach of the tip to the molecule. The concomitantly increased tip–molecule hybridization may be accompanied by electron transfer from the tip to the molecule reflecting the occupation of the MO and its shift toward and across *E*_F_. This rationale is in line with the predicted^[^ ^69^ ^]^ and experimentally observed^[^ ^70^ ^]^ different shifts of the C_60_ lowest unoccupied MO on various noble‐metal surfaces. The charge transfer may likewise be induced by the local work function difference between tip and molecule.^[^ ^71^ ^]^

## 3 Discussion and Perspectives to Future Studies

The presented finding of an electronic resonance at *E*_F_ enhancing AR can intuitively be understood as the improved low‐voltage transmission of the junction, i. e., as a lowered BTK barrier strength at the NC–SC interface. Conversely, moving the electronic resonance out of the Fermi level region gives rise to a deterioration of the junction transmission and, concomitantly, to the attenuation of AR. This picture of resonantly enhanced AR was obtained owing to observations from a molecular adsorbate with tunable electronic structure. It is, however, not obvious whether this mechanism is applicable to clean surfaces (Figure 2), too. Electronic resonances with externally controlled energy would be required to this end. Shockley surface states that are genuine to, e. g., the (111) surfaces of Ag, Cu, and Au, were previously shown to shift further below *E*_F_ upon approaching the STM tip owing to the concomitantly increasing electric field across the biased tunneling junction.^[^ ^72^, ^73^ ^]^ Unoccupied *p*‐type surface states of Pb(111) have indeed been predicted with a dispersion close to *E*_F_.^[^ ^74^ ^]^ Unlike the Shockley surface states of the coin metals that exhibit a parabolic dispersion around the $\bar{\Gamma }$‐point of the surface Brillouin zone, the dispersion of the low‐energy surface states of Pb(111) occurs in a surface‐projected energy gap of bulk states around the $\bar{K}$‐point. Therefore, their contribution to the tunneling current in an STM junction may be weakened or even entirely suppressed. For Nb(110), an occupied $4d_{z^{2}}$ surface resonance close to *E*_F_ with flat dispersion around the $\bar{\Gamma }$‐point and a strong signal in d*I*/d*V* spectra was identified.^[^ ^75^ ^]^ It will be interesting to see whether this resonance is subject to a shift toward *E*_F_ with tip approach, which would then explain the strong enhancement of AR observed in these contacts. Additional experiments that may likewise corroborate the picture of resonantly enhanced AR are listed below.

### 3.1 Spin Polarization of the Junction Current

The presence of an electronic resonance close to *E*_F_ affecting AR impacts the measurement of the current spin polarization using AR across magnetic nanoscale structures, such as single atoms or molecules. While for microscopic ferromagnet–SC interfaces AR was an appropriate means to access the spin polarization of the current across the junction,^[^ ^24^, ^25^, ^26^, ^27^ ^]^ AR in single‐atom or single‐molecule contacts may be subject to atomic or molecular orbitals close to *E*_F_, which would have to be considered for reliably extracting the spin polarization of the current. The latter becomes particularly appealing for magnetic‐atom or magnetic‐molecule contacts because a shifting orbital, atomic or molecular in nature, may contribute to the reduction of AR, which would otherwise be solely due to a spin‐polarized charge transport.^[^ ^24^, ^25^, ^26^, ^27^ ^]^ For Mn‐Pc on Pb(111) contacted with a W tip, the AR‐associated ZBR stayed below the maximum value of 2 · d*I*/d*V*|_*V* ≫ Δ/e_,^[^ ^76^ ^]^ which may hint at a possible spin polarization of the current or a shifting Mn‐Pc orbital.

Magnetic impurities further complicate the description because YSR states occur within the BCS energy gap^[^ ^66^, ^67^, ^68^ ^]^ due to the magnetic exchange interaction between the atomic or molecular magnetic moment and the Cooper pairs of the SC.^[^ ^77^, ^78^, ^79^, ^80^ ^]^ In addition, Kondo screening with the characteristic Abrikosov‐Suhl resonance^[^ ^81^, ^82^, ^83^ ^]^ at *E*_F_^[^ ^84^, ^85^, ^86^, ^87^ ^]^ may be present and contribute to the ZBR.^[^ ^88^ ^]^ It was previously demonstrated that YSR states induced by a Mn atom on Pb(111) can resonantly enhance AR even at low tunneling rates, i. e., at low junction transmission.^[^ ^89^ ^]^

### 3.2 Topological Superconductivity

The spectroscopic signature of topological superconductivity is the Majorana bound state, the condensed‐matter analog to the Majorana fermion.^[^ ^90^ ^]^ While the overall status quo in the search for this appealing quasiparticle state is summarized in recent review articles,^[^ ^91^, ^92^ ^]^ atomic or molecular spin chains on conventional superconductors represent particularly promising nanostructures for the fabrication of topological superconductivity and the Majorana bound state.^[^ ^93^, ^94^ ^]^ In these approaches to the Majorana bound state,^[^ ^95^, ^96^, ^97^, ^98^, ^99^, ^100^, ^101^, ^102^, ^103^, ^104^, ^105^, ^106^, ^107^ ^]^ the ferromagnetic coupling of the spins in the atomic chains polarizes the singlet Cooper pairs of the conventional superconductor leading to their triplet pairing, which represents an important ingredient for the Majorana quasiparticles occuring at the edges of the magnet–superconductor hybrid.^[^ ^108^ ^]^

A different approach to the Majorana quasiparticle was previously suggested by introducing superconductivity into a thin metal film on a conventional bulk superconductor.^[^ ^109^ ^]^ This theoretically predicted method was later approved experimentally.^[^ ^110^, ^111^, ^112^ ^]^ In short, the superconducting phase of V was introduced into a thin Au(111) film via the proximity effect. The main target was the achievement of superconductivity in the Shockley surface state band that exhibits a strong Rashba spin‐orbit splitting. The superconducting proximity effect was particularly efficient when the surface state was shifted close to the Fermi level, which was induced by the adsorption of EuS islands on top of the Au film.

The creation of superconducting correlations in surface states was likewise reported for Ag islands on Nb(110).^[^ ^113^ ^]^ In these experiments, the mechanical strain in the Ag islands was used to align the surface state energy with the Fermi level. The electron pairing was achieved by interband (surface state and bulk electron bands) coupling owing to a strong electron‐phonon interaction. Thin metal films on bulk superconductors become increasingly important for studies of, e. g., molecular spin excitation lifetimes^[^ ^114^ ^]^ or Cooper pair excitations in molecular junctions.^[^ ^115^ ^]^

The results of this Perspective outline a hitherto unexplored avenue to introduce Cooper pairs into a magnetic nanostructure. It relies on the optimized proximity effect induced by enhanced AR. To this end, the magnetic nanostructure is suggested to consist of an assembly of ferromagnetically coupled molecules that exhibit an orbital at the Fermi level. This orbital structure may chemically be engineered by considering a possible charge transfer upon adsorption. Another appealing possibility is the on‐surface tuning of the molecular assembly by the tip‐induced manipulation of matter. Indeed, it has recently been shown that electrostatic effects in artificial assemblies of 2H‐Pc molecules on Pb(100) can alter the energy of molecular orbitals.^[^ ^116^ ^]^ Magnetic molecular nanostructures on conventional superconductors therefore appear to be a viable route to topological superconductivity and the sought‐after Majorana bound state.

### 3.3 Andreev Reflection and Confined Electron Motion

To further corroborate the importance of electronic resonances at *E*_F_ for AR, different approaches to modifying the DOS at the Fermi level are proposed. Artificially fabricated atom corrals give rise to laterally confined electron states,^[^ ^117^, ^118^, ^119^, ^120^ ^]^ whose energies are controlled by the size of the quantum resonator. Tuning its dimensions by atom manipulation until a confined electron state spans the Fermi level region is anticipated to lead to enhanced AR compared to the situation where *E*_F_ lies in the gap between two successive quantized states. This approach has already been chosen in order to induce Machida‐Shibata states in the BCS energy gap of Nb(110) covered with Ag.^[^ ^121^ ^]^ The confinement of electrons is not restricted to corrals. Clusters, in particular linear chains of (magnetic) atoms, were successfully used to this end.^[^ ^122^, ^123^, ^124^, ^125^, ^126^, ^127^, ^128^, ^129^, ^130^, ^131^, ^132^, ^133^ ^]^

Another important example is electron confinement to quantum wells formed in ultrathin films on surfaces. Because the film–vacuum and the film–substrate interfaces act as barriers, electrons are confined vertically to the film giving rise to quantum well states, which are able to freely move in lateral directions.^[^ ^134^, ^135^ ^]^ Their importance for thin‐film SC^[^ ^136^, ^137^, ^138^ ^]^ and other electron correlation effects^[^ ^139^ ^]^ were previously demonstrated. For tuning the electron DOS at *E*_F_ in the case of a conventional SC, buried cavities on Pb(111)^[^ ^140^, ^141^ ^]^ may be particularly appropriate. These quantum resonators both laterally and vertically confine electron motion and give rise to eigenmodes with energies depending on their lateral size and depth beneath the surface.^[^ ^142^, ^143^, ^144^, ^145^, ^146^, ^147^ ^]^

### 3.4 Atomic‐Scale Engineering of Andreev Junctions

Experiments with an STM offer the unique opportunity to characterize the junction with atomic precision and to control its structural integrity prior to and after contact formation. Moreover, tip, substrate, and adsorbate material can be chosen at will. Therefore, model junctions comprising an atomically sharp tip, a pristine crystalline surface, and adsorbed atoms with clear‐cut atomic environment and with chosen orbital structure can be fabricated. Using the same tip for probing the *I*–*V* characteristics of the different atoms from tunneling to and beyond contact will open the path to associate the extent of AR with the atomic orbital structure. This knowledge may help establish a microscopic interpretation of the BTK model in the following sense. In the BTK approach to AR the systematic disentanglement of AR from the total conductance of an NC–SC junction with a very high number of electron transport channels, i. e., of a macroscopic NC–SC point contact, is achieved.^[^ ^18^, ^19^ ^]^ For atomic or molecular contacts, however, only a few electron transport channels are available, and the BTK picture is, at first sight, not applicable. Can the BTK model be used for each individual transport channel? First steps toward the elucidation of this question have already been taken, where the BTK barrier strength *Z* was related to the coupling of transport channels to the electrodes.^[^ ^47^ ^]^

## 4 Conclusion

This Perspective proposes the control of AR by electronic resonances straddling the Fermi level. Molecular orbitals are particularly suitable in this respect because their energy can be tuned by chemical engineering, hybridization with the electrodes or electrostatics. For nanotechnological applications, such as superconducting molecular electronics, the control of such resonances by an external electric field is therefore conceivable. In quantum physics, resonantly enhanced AR may optimize the proximity effect, which would be beneficial for the efficient injection of Cooper pairs into assemblies of magnetic atoms or molecules and the entailed study of triplet pairing or the fabrication of the sought‐after Majorana bound state. The general strategy followed in this Perspective is the spectroscopy of the BCS energy gap and the orbital electronic structure at tip–surface distances ranging from tunneling to and beyond contact. The thus enabled observation of evolving spectroscopic signatures upon gradually closing the vacuum barrier contains important information on the origin of zero‐bias anomalies in *I*‐*V* characteristics of junctions at the ultimate size limit.

## 5 Experimental Section

The experiments were carried out with an STM operated in ultrahigh vacuum (base pressure 5 · 10^−9^ Pa) and at low temperature (4.8 K). Constant‐height spectroscopy of d*I*/d*V* proceeded by the sinusoidal modulation (50 µV_rms_–3 mV_rms_, 360–3200 Hz) of the dc bias voltage applied to the sample and measuring the first harmonic of the ac current response of the junction with a lock‐in amplifier.

### Substrate and tip preparation

Clean crystalline (111) surfaces of Pb were prepared by Ar^+^ ion bombardment and annealing. The Nb(110) surface was prepared by repeated annealing between 2200 and 2650 K for various hours. Preparation of a Nb(110) crystal at these temperatures led to a reconstruction of the crystal surface due to the presence of oxygen, where the ideal body‐centered cubic structure of bulk Nb was covered by a single oxide layer of hexagonal NbO(111).^[^ ^148^, ^149^, ^150^, ^151^ ^]^ A single flash anneal between 2400 and 2650 K for 10 – 15 s was applied to reduce this oxide layer. Chemically etched W wire (purity 99.95 %) was used as the tip material.

### Molecule deposition

Phthalocyanine (2H‐Pc) and C_60_ molecules were sublimated from the solid phase (purity 98 %) in a heated (550 K) Ta crucible and deposited on clean Pb(111) at room temperature. Pyrrolic‐H‐abstracted phthalocyanine (Pc) was fabricated by the injection of high‐energy electrons from the STM tip across the central region of 2H‐Pc. To this end, the feedback loop was deactivated at 10 mV and 50 pA. The voltage was then ramped to 3 V for a duration of 2 s.

## Conflict of Interest

The authors declare no conflict of interest.
